# *MET* ‐amplified gastric cancers exhibit co‐amplifications of *BRAF* , *CDK6* , and *EGFR*

**DOI:** 10.1002/2056-4538.70104

**Published:** 2026-07-08

**Authors:** Silke Lüschen, Ulrike Ebert, Hans‐Michael Behrens, Sandra Krüger, Dita Ulase, Anu Amallraja, Steffen M. Heckl, Thomas Becker, Philip Rosenstiel, Tobias Meißner, Christoph Röcken

**Affiliations:** ^1^ Department of Pathology University Hospital Schleswig‐Holstein (UKSH), Campus Kiel Kiel Germany; ^2^ Department of Cancer Genomics Avera Cancer Institute Sioux Falls SD USA; ^3^ Department of Epidemiology & Biostatistics Memorial Sloan Kettering Cancer Center New York NY USA; ^4^ Department of Internal Medicine II, Hematology and Oncology University Hospital Schleswig‐Holstein (UKSH), Campus Kiel Kiel Germany; ^5^ Department of General Surgery, Visceral, Thoracic, Transplantation and Pediatric Surgery University Hospital Schleswig‐Holstein (UKSH), Campus Kiel Kiel Germany; ^6^ Institute for Clinical Molecular Biology Christian‐Albrechts‐University Kiel Germany

**Keywords:** gastric cancer, mesenchymal‐epithelial transition factor (MET), targeted therapy, co‐amplification, copy number variation

## Abstract

MET‐positive gastric cancer (GC) has a poor prognosis. In addition, targeting MET with monoclonal antibodies or tyrosine kinase inhibitors had limited success. We searched for additional genetic alterations that might explain the poor outcome of MET‐positive GC and lack of response to MET‐targeted therapies. Multiregional whole‐exome sequencing was performed on primary tumor and lymph node metastasis samples from two *MET*‐amplified discovery cases. Immunohistochemistry, *in situ* hybridization, and digital droplet polymerase chain reaction were performed in a validation cohort of 24 *MET*‐amplified GCs. Whole‐exome sequencing found 191 and 166 non‐synonymous mutations, respectively. Only eight genes showed non‐synonymous mutations in both cases, including *TP53*. Copy number variations (CNVs) were found in 18 and 14 autosomes, respectively, most commonly affecting chromosome 7. Since *BRAF*, *CDK6*, and *EGFR* are also localized on chromosome 7, validation studies were performed by fluorescence or chromogenic *in situ* hybridization, and co‐amplification was found in 54% of the cases, including two discovery and 24 validation cases. Different combinations were found, for example, *CDK6 + MET*, *EGFR + MET*, *CDK6 + EGFR + MET*, *BRAF + CDK6 + EGFR + MET*, or *BRAF + EGFR + MET*. A database search using cBioPortal confirmed our findings. However, marked intra‐ and intertumoral heterogeneity was observed, and CNVs were highly variable and spatially separated. *MET* amplification in GC is accompanied by amplification of other oncogenes located on chromosome 7, that is, *BRAF*, *CDK6*, and *EGFR*, in a highly heterogeneous manner leading to substantial intra‐ and intertumoral heterogeneity. This may cause diagnostic and therapeutic challenges, provoke subclonal evolution, and lead to poor prognosis of *MET*‐amplified GC and the failure of targeted therapies.

## Introduction

Gastric cancer (GC) is the third leading cause of cancer‐related death worldwide (Global Cancer Observatory, 2019; https://gco.iarc.fr/en). Its prognosis is usually poor, as the tumors are often only diagnosed at an advanced stage and the therapeutic options are then limited [1]. The only possibility of cure is resection of the tumor, with or without perioperative chemotherapy, depending on tumor stage [1]. Patients with advanced, inoperable GC or tumor recurrence now have access to several targeted drugs that are directed against tyrosine kinase receptors (trastuzumab) [2], ligands of tyrosine kinase receptors (ramucirumab) [3], immune checkpoint molecules (nivolumab, pembrolizumab) [4, 5], cell adhesion molecules (zolbetuximab) [6], neurotrophic tyrosine receptor kinase gene fusion (entrectinib, larotrectinib) [7]. There are only a few independent prognostic markers such as resection status, tumor regression grade according to Becker, nodal status, lymph node ratio, microsatellite instability, and particularly, the mesenchymal‐epithelial transition factor/hepatocyte growth factor receptor (MET) status [8]. Despite the successes with targeted drugs, the prognosis for GC remains poor. Particularly MET‐positive GCs exhibit a very poor prognosis.

MET is a tyrosine kinase receptor, which is overexpressed in 3.8–85% of GCs [8, 9]. We and others have shown that MET‐positive GCs, defined by a combination of immunohistochemical detection of moderate or strong membranous expression and *MET* amplification, have a very poor prognosis with a mean overall survival of less than 6 months [8, 10, 11]. MET‐positive tumors belong to the molecular subgroup of chromosomal instable GCs (CIN GC). These tumors are characterized by a high degree of chromosomal alterations, which frequently harbor also *TP53* mutations [12] and may not constitute a distinct subgroup but rather be a compilation of a more heterogeneous group of tumors [13].

Previous studies on the use of MET inhibitors, that is, monoclonal antibodies or tyrosine kinase inhibitors, in MET‐positive GCs have not been successful [9, 14, 15], so we hypothesized that the prognosis of MET‐positive GCs and hence the response to MET‐targeted therapy may not depend solely on MET status, but that there may be other prognosis and response determining genetic alterations that explain the poor outcome. To gain more information on the genetic characteristics, we performed whole‐exome sequencing (WES) on two chemotherapy‐naïve *MET*‐amplified discovery cases and validated our findings in a series of 24 *MET*‐amplified GCs. While only few overlapping non‐synonymous mutations (including *TP53* mutations) could be detected, both discovery cases were characterized by a high degree of intratumoral heterogeneity and showed co‐amplifications of several oncogenes mainly on chromosome 7, such as *BRAF*, *CDK6* and *EGFR*. Based on these findings we hypothesize that patient outcome of *MET*‐amplified GCs may also depend on the co‐amplification of other oncogenes located on chromosome 7.

## Materials and methods

### Study design

**Figure 1:**
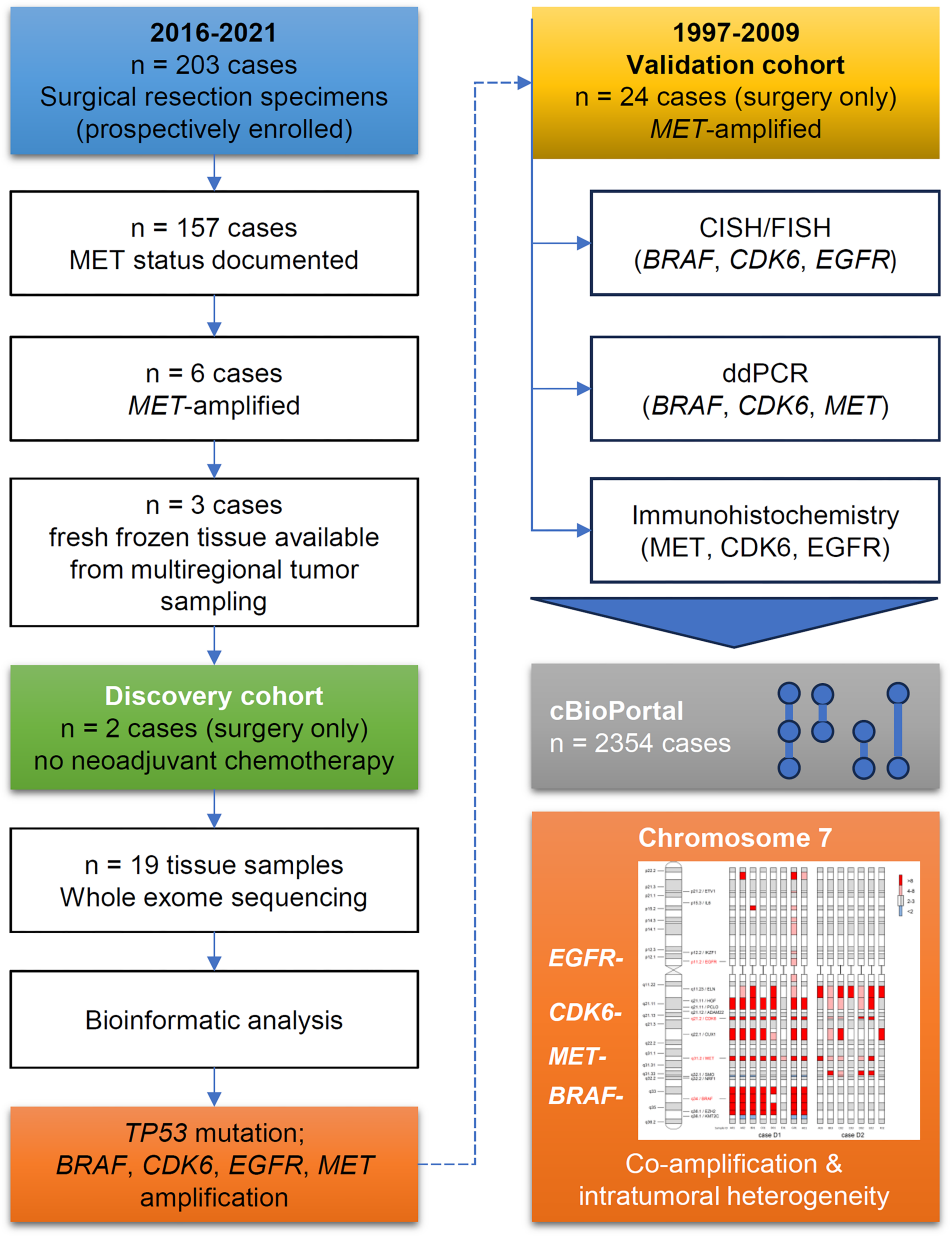
Flow chart of the study design.

The study design was exploratory and aimed to collect and analyze specimens. It did not involve randomization, blinding, replication, or power analysis, as the primary goal was to generate hypotheses and inform future studies. The study design is outlined in Figure 1.

### Study population

#### Discovery cases

Between 2016 and 2021, we prospectively enrolled 203 patients with an adenocarcinoma of the stomach or esophagogastric junction at the University Hospital Schleswig‐Holstein, Campus Kiel. All patients were Caucasian patients from Northern Germany treated in a single center. Immediately after the tumor was resected, the specimens were delivered on ice to the Department of Pathology. Depending on the size of the primary tumor, variable numbers of samples were punched out in a standardized manner using a core needle biopsy, and frozen at −80 °C in order to perform whole‐exome sequencing (see below). Among this prospectively collected cohort we than selected cases based on the MET status as assessed by immunohistochemistry and chromogenic *in situ* hybridization (CISH) [10]. The MET status was documented in 157 (77.3%) patients, of whom 6 (3.8%) cases were *MET*‐amplified. In three of these six cases, sufficient amounts of fresh frozen tissue were available (size of the primary tumor >3 cm) to enable multiregional tissue sampling and subsequent whole‐exome sequencing. In two of them no neoadjuvant chemotherapy had been administered prior to surgery (=chemotherapy‐naïve). In order to avoid a bias due to neoadjuvant chemotherapy, for example, clonal selection, we finally rendered these two cases suitable as discovery cases (Table 1). In these two cases, three unfixed fresh frozen samples had been obtained from each primary tumor (*n* = 6) and one sample from each corresponding non‐neoplastic mucosa (*n* = 2). In order to extend tissue sampling and to reduce the risk of a sampling error, we additionally collected formalin‐fixed paraffin‐embedded (FFPE) samples from the primary tumors (*n* = 2), lymph node metastases (*n* = 7), and non‐neoplastic mucosa (*n* = 2), which had been used for routine diagnostics. Finally, 15 tumor samples and 4 tissue samples of the corresponding non‐neoplastic stomach mucosa, that is, 19 tissue samples in total, were forwarded to whole‐exome sequencing (Table 1).

**Table 1:** Patient characteristics of the discovery cohort

	Case no. 1 (D1)	Case no. 2 (D2)
Sex (age)	Male (71 years)	Male (85 years)
Localization	Gastroesophageal junction	Gastroesophageal junction
Tumor type	Mixed type	Poorly differentiated
pTNM	pT3 pN3a (9/24) M0 L1 V1 Pn0 pR0	pT4a pN3a (8/21) pM1 (HEP) L1 V1 Pn1 pR1
**Whole‐exome sequencing**		
Primary tumor (fresh frozen)	3 samples	3 samples
Primary tumor (FFPE)	1 pooled sample form 6 tumor bearing paraffin blocks	1 pooled sample form 6 tumor bearing paraffin blocks
Lymph node metastasis (FFPE)	4 samples	3 samples
Non‐neoplastic control (fresh frozen)	1 sample	1 sample
Non‐neoplastic control (FFPE)	1 sample	1 sample
**Total number**	10 samples	9 samples

FFPE, formalin‐fixed paraffin embedded tissue.

#### Validation cohort

The validation cohort was collected from the archive of the Department of Pathology and had been part of a previous study on MET [10]. The validation cohort consisted of 24 patients (Table 2). All of these patients had undergone either a total or partial gastrectomy for adenocarcinoma of the stomach or esophagogastric junction between 1997 and 2009 and had been shown to be *MET*‐amplified based on immunohistochemistry and *MET*‐CISH [10]. None of these patients had undergone perioperative or neoadjuvant chemo‐ or radiotherapy (=chemotherapy‐naïve). All tissue samples originated from routine therapeutic surgeries, had been immediately formalin‐fixed paraffin‐embedded.

**Table 2:** Patient characteristics of the validation cohort

Case no.	Sex	Age (years)	Localization	Tumor type (Lauren)	pT	pN	pM	pL	pV	Pn	pR
V1	M	76	Distal stomach	Intestinal	T3	N2	M1	L1	V0	Pn1	R0
V2	F	65	Distal stomach	Intestinal	T4a	N3b	M1	L0	V1	Pn0	R0
V3	F	58	Distal stomach	Unclassified	T1b	N3b	M0	L1	V1	Pn0	R0
V4	F	60	Gastroesophageal junction	Intestinal	T3	N3a	M1	L1	V0	Pn1	R0
V5	F	58	Distal stomach	Diffuse	T4b	N2	M0	L1	V1	Pn1	R0
V6	M	83	Cardia	Diffuse	T4a	N3a	M0	L1	V0	Pn1	R1
V7	M	45	Cardia	Mixed	T3	N3a	M0	L1	V1	Pn1	R0
V8	M	64	Distal stomach	Diffuse	T3	N2	M0	L1	V0	Pn1	R0
V9	M	78	Distal stomach	Mixed	T4a	N2	M0	L0	V0	Pn1	R1
V10	F	56	Distal stomach	Diffuse	T4b	N3b	M1	L1	V0	Pn1	R1
V11	M	31	Distal stomach	Diffuse	T4a	N3a	M1	L1	n.a.	Pn0	R1
V12	F	72	Distal stomach	Diffuse	T4a	N3a	M1	L1	V0	Pn1	R0
V13	M	66	Distal stomach	Unclassified	T4a	N3b	M0	L1	V0	Pn1	R0
V14	M	73	Cardia	Intestinal	T3	N0	M0	L0	V0	n.a.	R0
V15	M	69	Distal stomach	Mixed	T4a	N2	M0	L1	V0	Pn1	R0
V16	M	52	Cardia	Mixed	T4a	N3a	M0	L0	V0	Pn1	R0
V17	M	79	Distal stomach	Intestinal	T2	N2	M0	L1	V0	Pn1	R0
V18	M	70	Gastroesophageal junction	Mixed	T3	N2	M0	L1	V1	Pn1	R1
V19	M	28	Distal stomach	Diffuse	T3	N3b	M1	L1	V0	Pn1	R1
V20	M	68	Cardia	Mixed	T4a	N3a	M1	L1	V0	Pn1	R1
V21	M	91	Cardia	Intestinal	T4a	N3b	M0	L1	V0	Pn1	R1
V22	M	63	Cardia	Intestinal	T4a	N3b	M0	L1	V1	Pn0	R0
V23	M	67	Gastroesophageal junction	Intestinal	T1b	N0	M0	L0	V0	Pn0	R0
V24	M	68	Distal stomach	Unclassified	T3	N3b	M1	L0	V0	Pn1	R1

F, female; M, male; n.a., not available.

### DNA sequence analysis by whole‐exome sequencing (discovery cohort)

Cryosections from fresh frozen core needle biopsies were stained with hematoxylin and eosin (H&E) prior to DNA isolation to guarantee tumor cell content. Genomic DNA was extracted from frozen tissue using the AllPrep DNA/RNA Mini Kit (Qiagen, Hilden, Germany). In addition, from FFPE tissue blocks, genomic DNA was extracted using the QIAamp DNA mini kit (Qiagen). Tissue sections were manually microdissected prior to DNA isolation to ensure a tumor cell content of higher than 80%. The integrity and amplifiability of the isolated DNA were evaluated by a qualitative size range PCR assay. DNA Exome libraries were prepared using the Illumina DNA Prep with Enrichment (Illumina, San Diego, CA, USA) in combination with the xGen™ Exome Hyb Panel v2 (IDT, Coralville, Iowa, USA). To exclude formalin fixation‐induced sequencing artefacts from FFPE material, we compared the sequencing data from two prepared libraries. Libraries were normalized, and sequencing was performed using 2 nM per library on a NovaSeq 6000 instrument (Illumina) with 1% phiX spike‐in at 2× 100 bp paired‐end settings. The purity‐ and ploidy status, and the sequencing statistics are shown in supplementary material, Table S1.

### Bioinformatic analysis

Primary data analysis [16], somatic mutation calling [17, 18, 19, 20, 21, 22, 23, 24, 25], copy number profiling [26], assessment of tumor mutation burden [27], microsatellite instability, and viral sequence analysis were done as described previously [28] (Supplementary Materials and Methods).

### Sanger sequencing

Sanger sequencing of *TP53* was performed as described previously [29].

### Histology

Tissue specimens used for histology and immunohistochemistry were fixed in formalin and embedded in paraffin. Deparaffinized sections were stained with H&E. Histological examination of primary tissue sections was carried out for all cases (discovery and validation cohort) to assure that inclusion criteria were met. Tumors were classified according to Lauren [30]. pTNM‐stage of all study patients was determined according to the eighth edition of the UICC guidelines [31].

### Immunohistochemistry

The procedures, the used antibodies and the evaluation of the staining results are described in Supplementary Materials and Methods.

### *In situ* hybridization

Amplification of *BRAF* and *CDK6* was assessed by fluorescence *in situ* hybridization (FISH) using the Zyto*Light*® SPEC BRAF/CEN7 Dual Color Probe Kit (ZytoVision, Bremerhaven, Germany, #Z‐2191‐200) and the CDK6/CCP7 FISH Probe Kit (CytoTest Inc., Rockville, USA, #CT‐PAC331‐10‐OG). Amplification of *EGFR* and *MET* was assessed by CISH using the Zyto*Dot*® 2C SPEC EGFR/CEN7 Probe and the Zyto*Dot*® 2C SPEC MET/CEN7 Probe, respectively, in combination with the Zyto*Dot*® CISH Implementation Kit (ZytoVision, #C‐3057‐400, #C‐3033‐400, and #C‐3044‐40). The results of FISH/CISH were evaluated by screening the entire tissue sections in order to find, where present, *BRAF*‐, *CDK6*‐, *EGFR*‐, or *MET*‐amplified areas of tumor cells. Subsequently, gene and centromere 7 signals were counted in at least 20 representative adjacent cancer cell nuclei within the tumor and the ratio of gene signals/centromere 7 signals was calculated. The presence of FISH/CISH clusters was noted. The gene count was calculated by dividing the number of gene signals by the number of cancer cell nuclei studied. Cases with a ratio >2.0 and cases with a ratio ≤2.0 but a gene count ≥6 were classified as amplified. In some cases, only a few adjacent nuclei showed amplification signals, so that counting of 20 nuclei resulted in values below the defined thresholds. These cases were marked with * and counted among the amplified cases (supplementary material, Table S8).

### Analysis of *BRAF* , *CDK*, and *MET* amplification by droplet digital polymerase chain reaction

The procedures, the primer sequences, the used assay kits, and the equipment are described in Supplementary Materials and Methods [32, 33].

### Comparison of copy number variation data with data from cBioPortal

The cBioPortal for Cancer Genomics was used to verify (co‐)amplifications of *BRAF*, *CDK6*, *EGFR*, and *MET* in public databases (date of analysis: July 16, 2024). The selected studies and the selected cancer types are described in Supplementary Material and Methods [12, 34, 35, 36, 37, 38, 39].

### Ethical statement

All executed procedures were in accordance with the ethical standards of the responsible committee on human experimentation (institutional and national) and with the Helsinki Declaration of 1964 and later versions. Prior to the respective procedures, all patients had given written informed consent for a possible future scientific use of their biological material. Ethical approval was obtained from the local ethical review board (D 453/10 and D 525/15) of the University Hospital Schleswig‐Holstein, Kiel, Germany. All patient data were pseudonymized after study inclusion. All experimental work complied with all mandatory laboratory health and safety procedures.

### Statistical analysis

All data are presented descriptively.

## Results

### Whole‐exome sequencing of two discovery cases: non‐synonymous mutations

The clinicopathological characteristics of the two discovery cases (D1 and D2) are shown in Table 1. A total of 19 tissue samples obtained from these two discovery cases were forwarded to WES (Table 1).

After WES, 191 (case D1) and 166 (case D2) non‐synonymous mutations (i.e., missense, nonsense and frameshift) were found with nine and six genes harboring ≥2 mutations (range 2–8), respectively (supplementary material, Tables S2–S4). The comparison of both cases showed that 23 genes were mutated in all tumor samples of case D1 and 66 genes were mutated in all tumor samples of case D2 (supplementary material, Table S5). Only eight genes showed non‐synonymous mutations in both cases, that is, *ACLY*, *FMNL2*, *GOLGA6L4*, *PLIN4*, *PRB3*, *TNFRSF10C*, *TP53*, and *ZNF497*. Among all tumor bearing samples analyzed, *TP53* mutations were found most commonly, that is, in 13 out of 15 samples (86.7%) with an allele frequency of 0.14 up to 0.57 and can thus be considered the only common denominator of non‐synonymous mutations in the two *MET*‐amplified discovery cases (supplementary material, Table S5). The *TP53* mutations (p.W146X in case D1 and p.R248Q in case D2) were validated by Sanger sequencing (supplementary material, Figure S1). Mutation of *TP53* is characteristic for chromosomal instable GCs with different types of tyrosine kinase receptor amplifications [12]. Thus, *TP53* mutations alone cannot explain the poor prognosis of *MET*‐amplified cases. Regarding the other seven genes, none was found to be mutated in the analysis from *The Cancer Genome Project* and therefore the other mutations were classified as case‐specific mutations without relevant significance for *MET*‐amplified GCs [12]. No *BRAF* mutation (including p.V600E) was found in the discovery cases (supplementary material, Table S2). Thus, no non‐synonymous mutation could be identified that would explain the poor prognosis.

### Whole‐exome sequencing of the discovery cases: copy number variations

We then examined the copy number variations (CNVs) in the two discovery cases. CNVs were found in 18 and 14 autosomes, respectively, most commonly affecting chromosome 7 (supplementary material, Tables S6 and S7). The number of CNVs in the autosomes varied considerably between the two cases and also among different samples within each case, affecting 142 (case D1) and 43 genes (case D2). Between 0.7% and 4.7% of the CNVs were found in all samples of the same patient.

Comparing the two cases, 128 autosomal genes showed CNVs only in case D1 and 29 genes only in case D2. Fourteen autosomal genes showed CNVs in both cases, that is, *AKAP9*, *ARHGEF10L*, *CDK6*, *CUX1*, *ELN*, *FUS*, *GRM3*, *HIP1*, *MEN1*, *MET*, *PAX7*, *RECQL4*, *SMO*, and *TRRAP*. Among all 15 tumor bearing samples, *MET* (13/15), *AKAP9* (11/15), *GRM3* (11/15), *HIP1* (11/15), *CUX1* (10/15), *ELN* (9/15), and *CDK6* (8/15) were most frequently altered and all located on chromosome 7. Further details of the sequencing data are provided in supplementary material, Tables S6 and S7.

Taken together, these data from two *MET*‐amplified discovery cases illustrate the genetic complexity of interindividual and intratumoral heterogeneity affecting both single nucleotide variations and CNVs.

### Intertumoral heterogeneity of *BRAF* ‐, *CDK6‐*, *EGFR* *‐*, and *MET* amplification in gastric cancer

**Figure 2:**
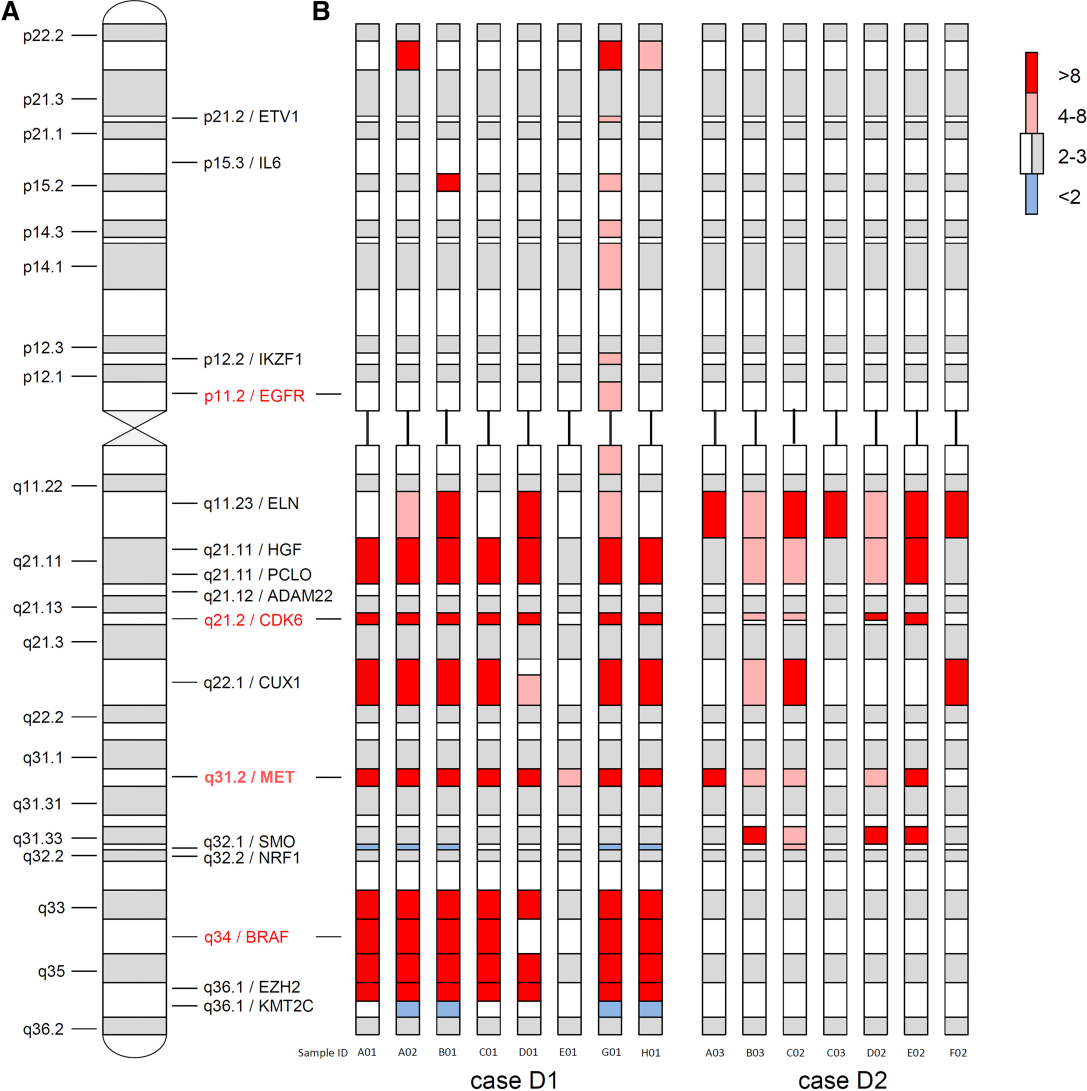
Graphical illustration of the chromosomal localization of the four oncogenes studied here (A) and illustration of intratumoral heterogeneity of copy number variations in the samples of the two discovery cases (B). BRAF, b‐Raf proto‐oncogene; CDK6, cyclin dependent kinase 6; EGFR, epidermal growth factor receptor; MET, MET proto‐oncogene.

Interestingly, in addition to *MET* amplification both discovery cases also showed co‐amplifications of other oncogenes localized on chromosome 7, that is, *BRAF, CDK6*, and *EGFR* (supplementary material, Tables S6 and S7), whose negative prognostic impact in GC has already been shown [40, 41]. Thus, at least four putative oncogenes are localized on chromosome 7 (Figure 2), and the findings from the two discovery cases raised the possibility of general co‐amplification of these oncogenes in *MET*‐amplified cases. To test this hypothesis, we increased the number of cases by adding 24 *MET*‐amplified GCs (validation cohort; cases V1–V24). Representative FFPE tissue blocks from each of the 24 validation cases and the two discovery cases were selected and subjected to CISH (*EGFR*) or FISH (*BRAF*, *CDK6*). Lymphocytes, non‐neoplastic epithelia, smooth muscle cells, and stromal cells were used as internal negative controls, each with a wild‐type set of ISH signals.

**Figure 3:**
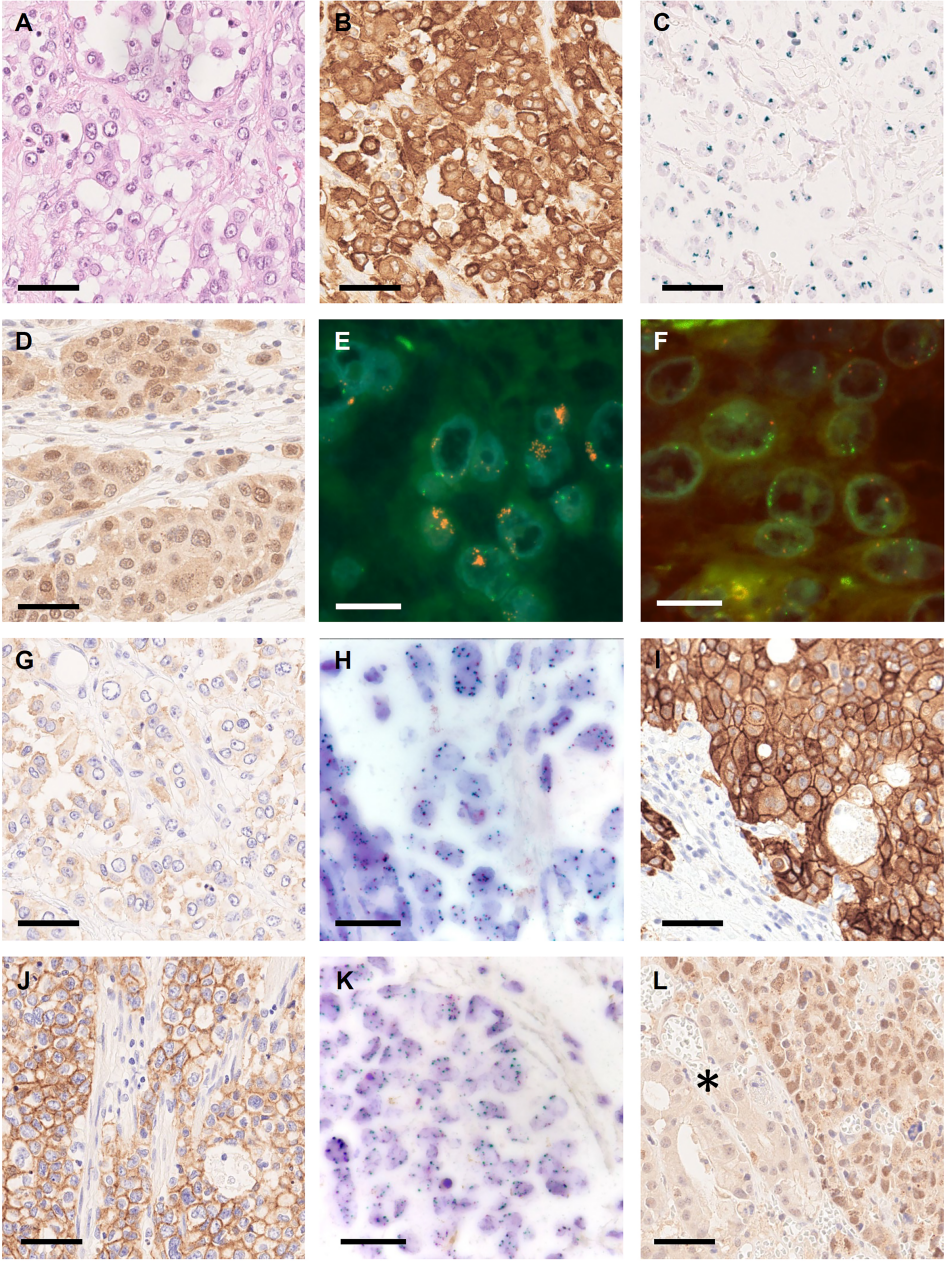
Gene amplification and protein expression assessed by immunohistochemistry (IHC; B, D, G, I, J, L), chromogenic (CISH; C, H, K), or fluorescence *in situ* hybridization (FISH; E, F). One of two discovery cases (D1) with a MET‐positive gastric cancer (A–H). Hematoxylin and eosin‐staining (A). The tumor showed areas with strong membranous expression of MET (B), *MET* amplification (C), moderate to strong nuclear expression of CDK6 (D), *CDK6* amplification (E), *BRAF* amplification (F), weak expression of EGFR (G), and *EGFR* amplification (H). One of the cases of the validation cohort (V7) (I–L) showed a strong membranous expression of MET (I) and of EGFR (J), *EGFR* amplification (K), and moderate to strong nuclear expression of CDK6 in the tumor cells and unstained non‐tumor tissue (asterisk; L). Original magnifications: ×400 (A–D, G, I, J, L), ×1,000 (E, F, H, K). Scale bar indicates 50 μm. BRAF, b‐Raf proto‐oncogene; CDK6, cyclin dependent kinase 6; EGFR, epidermal growth factor receptor; MET, MET proto‐oncogene.

**Figure 4:**
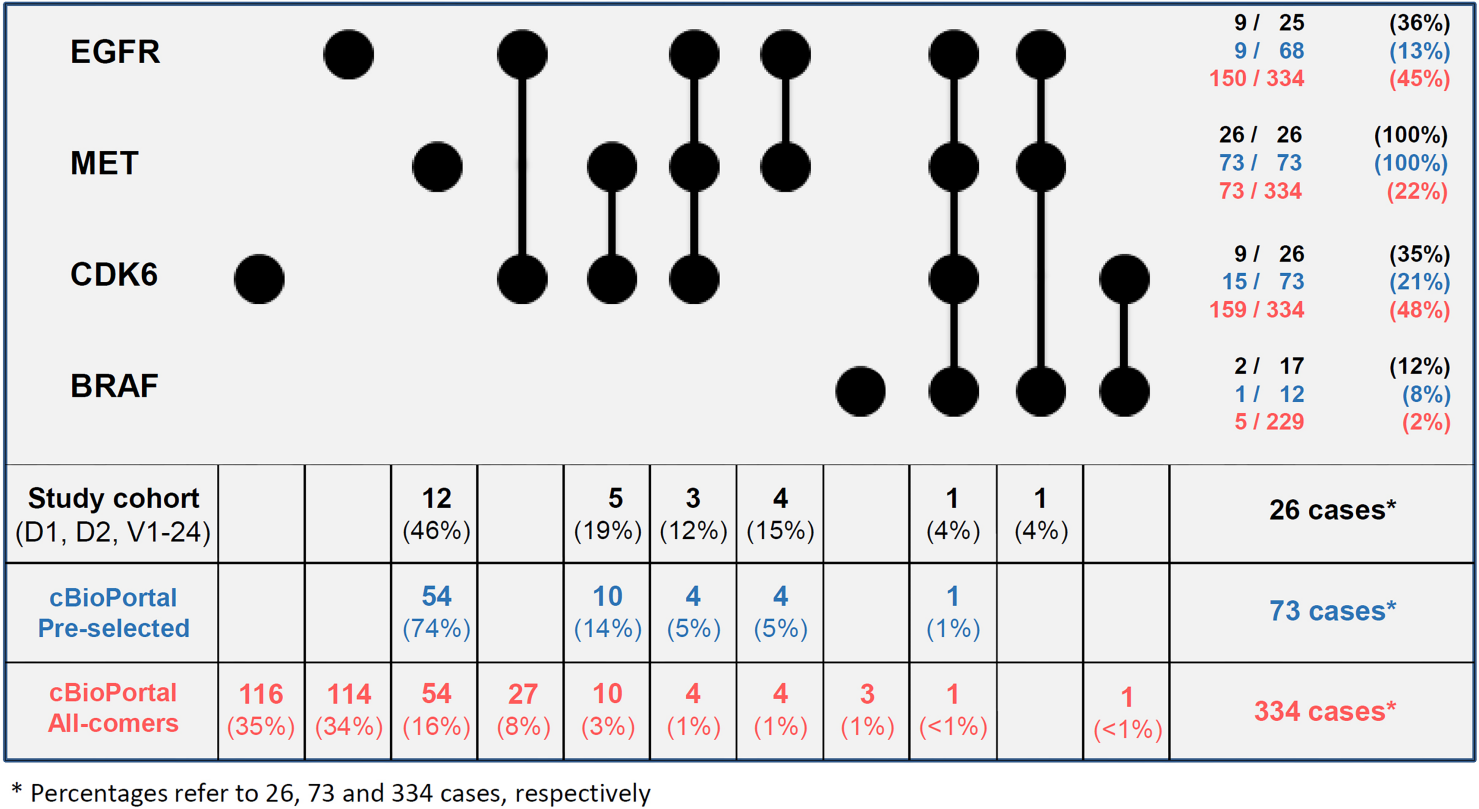
UpSet plot illustrating the frequency and distribution of (co‐)amplifications of *BRAF*, *CDK6*, *EGFR*, and *MET* in adenocarcinomas of the esophagogastric junction and stomach. Data from our study (black letters; *n* = 26 cases) were compared with two data sets obtained from cBioPortal, that is, *MET*‐amplified cases (pre‐selected; blue letters; *n* = 73 cases) and cases with amplification of any of the four genes (all‐comers; red letters; *n* = 334 cases). BRAF, b‐Raf proto‐oncogene; CDK6, cyclin dependent kinase 6; EGFR, epidermal growth factor receptor; MET, MET proto‐oncogene.

As shown in Figure 3 and summarized in Figure 4 co‐amplification of two, three, and four oncogenes, respectively, assessed by CISH/FISH occurred in 54% of our cases, including the two discovery cases and the 24 validation cases. Different combinations were found, that is, *CDK6 + MET* (5 cases), *EGFR + MET* (4), *CDK6 + EGFR + MET* (3), *BRAF + CDK6 + EGFR + MET* (1), and *BRAF + EGFR + MET* (1). Accordingly, interindividual heterogeneity of gene amplification was observed for all four genes and showed no systematic pattern. Usually, there was no obvious spatial relationship of the co‐amplified genes. *BRAF* amplification was not associated with a *BRAF* mutation, for example, p.V600E (*BRAF* genotype data were available from a previous study) [42].

To independently validate our findings, we then performed a database search using cBioPortal (see Supplementary Materials and Methods for details). Eight databases were selected covering 2,354 patients with gastric or gastroesophageal junction adenocarcinoma. Among these cases, 73 (3%) patients harbored a *MET* amplification (Figure 4 – pre‐selected), of which 19 patients (26%) showed co‐amplification of *BRAF* (*n* = 1), *CDK6* (*n* = 15), and/or *EGFR* (*n* = 9) (Figure 4).

Given the considerable interindividual heterogeneity of the four selected oncogenes, we expanded our search criteria to identify any amplification of any of the four genes in gastric or gastroesophageal junction adenocarcinoma (Figure 4 – all‐comers). This resulted in the identification of 334 of all 2,354 patients with amplification of at least one of the considered oncogenes (14%). Of these 334 cases, 48% exhibited amplification of *CDK6*, followed by 45% for *EGFR*, 22% for *MET*, and 8% for *BRAF*. Co‐amplifications occurred in different combinations in 47 of the 334 (14%) cases (Figure 4).

### Correlation of *BRAF* , *CDK6*, and *MET* amplification assessed by *in situ* hybridization with digital droplet PCR

The higher percentage of co‐amplified cases in our patient cohort (54%) compared to the *MET* co‐amplified cases from the cBioPortal search (26%) raised the question of possible reasons for these different numbers. Light and fluorescence microscopic evaluation of gene amplification allows the detection also of a small proportion of amplified tumor areas (basically on a single cell level) and therefore has a high sensitivity. And indeed, we were able to detect intratumoral heterogeneity for all genes as well, sometimes showing only small tumor areas with gene amplification (less than 10% of the tumor cells). So, there is definitely a risk of a false low value in the cBioPortal cases, and co‐amplification is much more common than the database search suggests. To support this hypothesis, we tested the (co‐)amplification data from *in situ* hybridization analysis using an independent method.

We chose ddPCR as the second validation method. To avoid misinterpretation of polysomy, we did not select the centromere region of chromosome 7 as a reference, but the *RPP30* gene on chromosome 10. For *CDK6*, the detected ratio in the non‐tumor controls ranged from 0.75 to 1.34 (median 0.86), comparable to the calculated ratio (0.7–1.48) from six (66%) of the nine *CDK6*‐amplified cases detected by FISH, including one of the discovery cases (D2). In three (33%) out of nine FISH‐detected cases, *CDK6* amplification was confirmed by ddPCR (D1, V3, and V7). For *BRAF*, the non‐tumor controls ranged from 0.95 to 1.15 (median 1.05), while the ratio detected in tumors ranged from 0.7 to 2.14. *BRAF* amplification was confirmed by ddPCR in two cases (D1, V16) and detected in three additional cases with a slight elevated ratio of 1.6 (V3), 1.75 (V10), and 1.58 (V13). *MET* amplification was confirmed by ddPCR in 10 (38%) out of 26 cases (ratio 2.55–13.8). In 16 cases, the calculated ratio in the tumor (0.36–1.75) was the same as in the non‐tumor samples (0.41–1.46, median 0.7) (supplementary material, Figure S2). Thus, ddPCR also detected co‐amplifications of *BRAF + CDK6 + MET* (*n* = 2, D1 and V3), *BRAF + MET* (*n* = 2, V13 and V16), and *CDK6* + *MET* (V7) (supplementary material, Figure S2) but could not confirm all amplifications detected by FISH/CISH analysis.

### Intratumoral heterogeneity of *CDK6* and *MET* copy number variation in gastric adenocarcinoma

While the previous results provided evidence for interindividual heterogeneity of (co‐)amplifications of the four oncogenes, we next wanted to investigate whether this also applies to individual cases (=intratumoral heterogeneity). To this end, we selected 17 tumor‐bearing paraffin blocks and 2 non‐tumor blocks from two *CDK6* and *MET* co‐amplified cases (D1 and V7). One of these two cases (D1) also showed a *BRAF* amplification, although to a lesser extent than *MET* and *CDK6* amplifications. In total, 32 microdissected samples from the 19 donor paraffin blocks [8 non‐tumor samples (NTS) and 24 tumor samples including MET‐positive and MET‐negative tumor areas (TS); supplementary material, Figure S3], were forwarded to ddPCR. Again, we observed a high degree of heterogeneity regarding gene amplification for *BRAF, CDK6*, and *MET* in the different tumor locations (supplementary material, Figure S3). Interestingly, in three MET‐negative tumor areas of case D1, amplification only for *CDK6* but not for *MET* or *BRAF* was detected (P4 with TS4 and TS13, P5 with TS5 and TS14, P6 with TS6 and TS15; supplementary material, Figure S3). In the internal negative controls (NTS) no *CDK6*, *BRAF*, or *MET* amplification was found.

While digital droplet PCR can be used to detect gene amplifications, it carries the risk of a false negative result. This phenomenon can be attributed to the presence of non‐neoplastic cells, such as desmoplastic stromal cells, inflammatory cells, and smooth muscle cells within the microdissected tumor area, which, despite being present in relatively low numbers, can significantly impact the analysis. Consequently, this can result in an inaccurate assessment of the gene copy number in the tumor cells, leading to an underestimation of the true value. Nevertheless, these data show a large interindividual and also intratumoral heterogeneity of chromosome 7 alterations.

### Immunohistochemical detection of MET, CDK6, and EGFR expression

**Figure 5:**
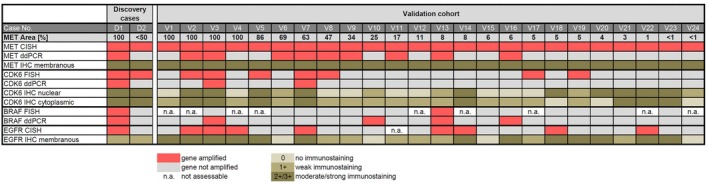
Case‐wise summary of the phenotypic and genotypic co‐alterations associated with *MET* amplification in two cases of the discovery cohort (D1, D2) and 24 cases of the validation cohort (V1–V24). BRAF, b‐Raf proto‐oncogene; CDK6, cyclin dependent kinase 6; CISH, chromogenic *in situ* hybridization; ddPCR, digital droplet PCR; EGFR, epidermal growth factor receptor; FISH, fluorescence *in situ* hybridization; MET, MET proto‐oncogene; n.a., not assessable.

Using the 2 cases from the discovery cohort and 24 cases from the validation cohort we then correlated the amplification status of *MET*, *CDK6*, and *EGFR* with their respective protein expression by immunohistochemistry (Figure 3). Ten (38%) cases showed moderate/strong nuclear and cytoplasmic staining for CDK6. In two (8%) additional cases (V18 and V20), moderate/strong staining was confined only to the nuclei. In all of these cases, moderate/strong CDK6 expression coincided with MET overexpression, although not spatially. Six (50%) of 12 cases with moderate/strong CDK6 expression also showed *CDK6* amplification (Figure 5), but in three cases with *CDK6* amplification, CDK6 expression was weak or absent. In contrast, six cases with moderate/strong CDK6 expression showed no gene amplification either by FISH or by ddPCR (Figure 5; supplementary material, Figure S4).

Regarding EGFR, intratumoral expression of EGFR was always heterogeneous. Thirteen (50%) cases showed moderate/strong membranous expression, being detectable in 5–90% of the tumor cells. In seven of these cases, *EGFR* amplification was found by CISH and in the other six cases no *EGFR* amplification could be detected (Figure 5; supplementary material, Figure S4). Conversely, two *EGFR*‐amplified cases showed only a weak expression of EGFR (supplementary material, Figure S4). For example, in the case of CDK6 and EGFR, we found that gene amplification did not always correspond to protein expression, and transcriptional or translational processes may affect the expression status of the corresponding protein.

## Discussion

*MET*‐amplified GCs have a very poor prognosis regardless of the percentage of *MET*‐amplified tumor cells [8, 10, 11]. Targeted therapies with antibodies to block MET‐dependent signaling were less successful [9, 14, 15]. This is in contrast to targeted therapies against other tyrosine kinase receptors like trastuzumab in HER2‐positive GC and also to other MET‐positive cancers like lung cancer where MET‐targeted therapies are more effective [43]. These observations led to the hypothesis that in *MET*‐amplified GC it is not MET alone that determines prognosis and treatment efficacy, but other, yet unassigned genetic alterations.

Gastric cancer exhibits a high degree of intratumoral genetic and translational heterogeneity, which in consequence carries a high risk of sampling error [28, 44, 45]. To address this issue and reduce the risk of sampling error, we performed whole‐exome sequencing comparing eight and seven tumor‐bearing tissue samples obtained from gastrectomy specimens of two *MET*‐amplified discovery cases with tissue samples of corresponding non‐neoplastic gastric mucosa. The results of the sequencing study confirm the presence of intratumoral heterogeneity, which is evident in both non‐synonymous mutations and CNVs. Regarding SNV, only eight genes including *TP53* were mutated in both discovery cases and surprisingly none could explain the poor prognosis of *MET*‐amplified GCs.

Therefore, we next focused on CNVs and it was then interesting to note that in addition to *MET*, three other putative oncogenes localized on chromosome 7, namely *BRAF*, *CDK6*, and *EGFR* (see Figure 1), could be co‐amplified, albeit with substantial variability. Although assessing the prognostic impact of these co‐amplifications was beyond the scope and design of our study, it raised several interesting issues regarding diagnostics and patient management.

Intratumoral heterogeneity impacts tissue‐based diagnostics. The *European Society for Medical Oncology (ESMO)* guidelines recommend that multiple (5–8) biopsies should be carried out to provide adequately sized material for histological and molecular interpretation [46]. Our data underscore the sensitivity of tissue sampling to detect co‐amplifications that may go undetected when only a single tumor sample (=biopsy) or a single paraffin block selected from a resection specimen is analyzed. And based on our findings and observations described by others, random sampling from multiple sites of the primary tumor and even metastatic organ sites may be strongly advised [44]. Methodological limitations further impede detection of gene amplifications. Tissue architecture with a mixture of tumor cells and surrounding non‐tumor and inflammatory cells might decrease the sensitivity of DNA‐based assays. As an example, using ddPCR we were not able to confirm the CISH detected *MET* amplification in all cases although we isolated the DNA from microdissected, immunohistochemically MET‐positive tumor areas. For these reasons even highly sensitive PCR methods (which ultimately also include NGS methods) might fail to detect (co‐)amplifications and the absence of evidence for gene amplifications does not unequivocally exclude their presence. Similar observations were made in non‐small cell lung cancer where the concordance rate among FISH and NGS to detect *MET* amplification was only 62.5% [47]. FISH analysis is considered to be the gold standard to detect gene amplification and offers in addition the possibility to discriminate between amplification by polysomy and focal amplification [48, 49]. Both the analysis of an insufficient number of tissue samples and the chosen detection method compromise tissue‐based diagnostics and miss (co‐)amplifications of tumor oncogenes that potentially affect patient prognosis, both synergistically and separately.

In terms of tumor biology, treatment response and patient prognosis, our data highlight the complexity and likely plasticity of CIN GCs. Co‐amplification of one of the other oncogenes located on chromosome 7 might prevent the therapeutic effect of standard chemotherapies or targeted therapies or even might lead to a selection pressure that promotes tumor cell clones with amplification of one of the other oncogenes [44]. Similar observations have been made in lung cancer in regard to EGFR targeted therapy: both *CDK4/6* and *MET* amplifications are linked to *de novo* EGFR tyrosine kinase inhibitor resistance [50, 51, 52]. Thus, affecting therapeutic response by amplification of chromosome 7‐located genes has been observed in other tumor entities, and it is tempting to speculate that this also applies to CIN GC. Co‐amplification of other tyrosine kinase receptors not located on chromosome 7, such as *ERBB2*, has been described and may also affect therapeutic response to MET inhibitors [10, 44]. However, they do not explain the therapy‐independent very poor prognosis of *MET*‐amplified GCs. This complex situation might explain why trials attempting to block MET signaling have not been successful, and why the majority of these trials have also not been successful in using MET‐based biomarker analysis to predict patient outcome [9, 15].

The results of our study have further important implications. We show that a single‐gene approach (i.e., a genetic alteration is assessed independent of the genetic context), as used in many clinical trials, and also molecular tumor boards, leads to misjudgements. In particular, in the case of CNV it is therefore important to consider, which other genes are located on the affected chromosome and whether there are any co‐amplifications that need to be taken into account in terms of tumor biology and therapy. If targeted drugs are to be used, the therapy would have to attack several oncogenes simultaneously in these special cases, otherwise there is a risk of promoting clonal selection in favor of the other oncogene(s) [53].

We suspect that MET‐positive GCs already show progression under neoadjuvant chemotherapy and are therefore no longer operated on. The collected cases in the validation cohort (25 *MET*‐amplified cases out of 470 cases in the period from 1997 to 2009) [10] were from the time before the introduction of perioperative chemotherapy and, as mentioned above, the search for discovery cases has taken a long time as MET‐positive GCs have become a rarity in surgical resection specimens in the era of perioperative chemotherapy (Röcken, C., personal observation). MET‐positive GC should possibly not even be included in a ‘curative standard therapy approach’, but must be assessed as putative palliative from the outset or should be treated with new combinatorial therapies. If we expand the previous focus on MET as a marker of a poor prognosis with a median survival of less than 6 months, we must broaden our view to other oncogenes located on chromosome 7 and check whether one of the four oncogenes investigated here is amplified.

### Limitations

Our study has several limitations. We do not provide mechanistic insights, and we did not specifically analyze downstream targets and pathways. Given the overall small sample size due to the overall rarity of *MET* amplification, we were also unable to assess any putative prognostic or therapeutic impact of co‐amplification(s). However, the primary goal was to generate hypotheses and inform future studies, to consider our findings for the design of future clinical trials.

## Conclusion

To the best of our knowledge, our study is the first systematic investigation of *MET*‐amplified tumors using multiregional whole‐exome sequencing. We reduced the risk of sampling error by using multiple (multiregional) tissue samples from the primary tumors and from lymph node metastases. We are the first to have conducted extensive validation studies including a series of 24 *MET*‐amplified GCs, used independent methods and cBioPortal data to identify methodological and technical issues and to highlight problems of heterogeneity. While only a few overlapping non‐synonymous mutations (including *TP53* mutations) could be detected, both discovery and validation cases showed co‐amplifications of several oncogenes located on chromosome 7. We are the first to explicitly point out that when an oncogene amplification on chromosome 7 is detected, other oncogenes located on this chromosome may also be amplified, conferring a high degree of plasticity on the tumor for the development of resistance. Our findings may help to develop new combination chemotherapies for these tumors which might be much more common than has been anticipated. For example, in previously treated metastatic gastroesophageal cancer, the CDK4/6 inhibitor palbociclib was less successful as a monotherapy [54], but could be tried in combination with anti‐MET targeted therapies in *MET*‐amplified GC. The same may be true for treatment with the CDK4/6 inhibitor abemaciclib [55]. Prospective clinical trials focusing on GC with chromosome 7 amplifications should take these data into account to further improve treatment efficacy and patient outcomes. However, adequate tissue sampling and methodological limitations must be considered.

## Author contributions statement

SL: data curation, formal analysis, validation, investigation, visualization, methodology, writing – original draft, writing – review and editing. UE: data curation, formal analysis, investigation, writing – review and editing. H‐MB: data curation, formal analysis, validation, writing – review and editing. SK: formal analysis, methodology, writing – review and editing. DU: validation, writing – review and editing. AA: data curation, software, formal analysis, investigation, writing – review and editing. SMH: data curation, investigation, writing – review and editing. TB: resources, data curation, writing – review and editing. PR: conceptualization, resources, project administration, writing – review and editing. TM: conceptualization, resources, data curation, software, formal analysis, visualization, methodology, project administration, writing – review and editing. CR: conceptualization, resources, data curation, formal analysis, supervision, funding acquisition, visualization, methodology, writing – original draft, project administration, writing – review and editing.

## Supporting information


Supplementary materials and methods
**Figure S1.** *TP53* mutations in the two discovery cases
**Figure S2.** Intertumoral heterogeneity of gene amplification(s): evaluation of gene amplification in two cases of the discovery cohort and 24 cases of the validation cohort of *MET*‐amplified gastric cancer assessed by digital droplet PCR
**Figure S3.** Intratumoral heterogeneity of gene amplification(s). Samples obtained from 17 tumor bearing and 2 non‐tumor bearing paraffin blocks obtained from 2 cases (D1 and V7) were forwarded to digital droplet PCR
**Figure S4.** Graphical illustration of the correlation of immunostaining with gene amplification for *CDK6* and *EGFR*

**Table S1.** List of purity‐ and ploidy status of the two discovery cases
**Table S2.** List of non‐synonymous mutations found in the two discovery cases
**Table S3.** List of non‐synonymous mutations found in the discovery case D1
**Table S4.** List of non‐synonymous mutations found in the discovery case D2
**Table S5.** List of non‐synonymous mutations found in both discovery cases (yellow) and in all samples of each discovery case (orange and blue)
**Table S6.** List of copy number variations found in the discovery case D1
**Table S7.** List of copy number variations found in the discovery case D2
**Table S8.** FISH and CISH signal counts and data analysis

## Data Availability

All data generated or analyzed during this study from all gastric cancer patients are included in this published article and its supplementary material. Raw sequencing data are available at the European Genome Archive (submission number: EGA50000000697).
