# Supplementary material for: MET ‐amplified gastric cancers exhibit co‐amplifications of BRAF , CDK6 , and EGFR
Source: J Pathol Clin Res. 2026 Jul 8;12(4):e70104. doi: 10.1002/2056-4538.70104 (PMC13343299; doi:10.1002/2056-4538.70104)
Supplement: Supplementary file 1 — Supplementary materials and methods Figure S1. TP53 mutations in the two discovery cases Figure S2. Intertumoral heterogeneity of gene amplification(s): evaluation of gene amplification in two cases of the discovery cohort and 24 cases of the validation cohort of MET‐amplified gastric cancer assessed by digital droplet PCR Figure S3. Intratumoral heterogeneity of gene amplification(s). Samples obtained from 17 tumor bearing and 2 non‐tumor bearing paraffin blocks obtained from 2 cases (D1 and V7) were forwarded to digital droplet PCR Figure S4. Graphical illustration of the correlation of immunostaining with gene amplification for CDK6 and EGFR [file CJP2-12-e70104-s002.pdf]

MET-amplified gastric cancers exhibit co-amplifications of *BRAF*, *CDK6*, and *EGFR*

S Lüschen, T Meißner, C Röcken *et al*. *J Pathol Clin Res*

https://doi.org/10.1002/2056-4538.70104

Supplementary Materials and Methods

Supplementary References

Supplementary Figures S1–S4

[NB: Supplementary Tables S1–S8 in separate Excel File]

Supplementary materials and methods

DNA sequence analysis by whole-exome sequencing (discovery cohort)

Primary data analysis

Raw fastq data were quality-trimmed, and adapter sequences were removed using bbduk from the BBTools suite version 36.32 (http://sourceforge.net/projects/bbmap; access date: August 2022 to June 2023) with the following parameters: minlen=25 qtrim=rl trimq=10 ktrim=r k=25 mink=11 hdist=1 overwrite=true tbo=t tpe=t. The Burrows-Wheeler aligner 0.7.15 (https://arxiv.org/abs/1303.3997; access date: August 2022 to June 2023) with default parameter settings was used to align the sequencing reads to the human reference genome (hs37d5). Duplicate reads were marked with sambamba 0.6.3 [56] and indel realignment was performed using ABRA version 0.97 [57]. FastQC 0.11.5 (http://www.bioinformatics.babraham.ac.uk/projects/fastqc/; access date August: 2022 to June 2023) and Qualimap 2.2 [16] were used to perform quality checks on the fastq and bam files respectively.

Somatic mutation calling

Paired tumor-normal variant calling was performed using VarDict 1.5.1 [17] with the following parameters: mapping quality Q=10, base quality phred score q=20, minimum allele frequency f=0.01, and number of nucleotides to extend for each segment x=2000. Additionally, the read position filter P=0.9 and maximum number of reads with mismatches m=4.25, were supplied to var2vcf_paired.pl with further downstream filtering steps to improve detection of low frequency variants as described by Brad Chapman (http://bcb.io/2016/04/04/vardict-filtering/; access date: August 2022 to June 2023). ANNOVAR [18] was used to annotate variants utilizing refGene, cosmic84, clinvar_20170905, icgc21, nci60, exac03, exac03nontcga, snp142, avsnp150,

1000g2015aug_all, ljb26_all, dbnsfp33a, and intervar_20180118 databases. Homopolymer regions were marked using vcfpolyx from the jvarkit suite (https://github.com/lindenb/jvarkit; access date: August 2022 to June 2023). Variants were retained if the following criteria were met: allele frequency (AF) ≥5%, total read depth ≥10 in either the tumor or the normal sample, variant depth in tumor sample ≥2, no strand bias, AF>10% in homopolymer regions. Variants in blacklisted regions described by Fuentes et al. [19] as well as ENCODE [20] were filtered out. In addition, annotation-based filtering was utilized to retain only coding, non-synonymous variants with ExAC AF <0.01 [21]. Variants unknown to either COSMIC or ICGC were retained only if they occurred within one of the 719 genes of the COSMIC Cancer Gene Census (https://cancer.sanger.ac.uk/census, downloaded June 12, 2018) [22] (https://dcc.icgc.org/; access date: August 2022 to June 2023). An additional variant recovery process on the list of filtered variants was implemented on a per patient basis to recover variants from individual samples with AF <5% if they were present in at least one other sample of the same patient with AF ≥5%. Tumor purity and ploidy were computationally estimated using Sequenza [23]. Clonality and cancer cell fraction (CCF) for each variant was determined using Palimpsest [24]. In brief, CCF is computed by adjusting the variant allele fraction for the tumor purity and the absolute copy number at each locus in tumor and normal cells. Mutations were classified as subclonal if the upper boundary of the 95% confidence interval was below the threshold of 0.95. COSMIC Mutational Signatures Version 2 were inferred using the R package deconstructSigs [25].

Copy number profiling

Allele-specific copy number calling was done using CNVkit 0.9.5 [26]. A pooled normal reference was created from the matched non-neoplastic stomach mucosa samples. The initial segments were called with a conservative significance threshold of t = 1e-6 and low coverage segments were dropped. Segments were then used along with raw variant calls from VarDict, the estimated tumor purity, to call major and minor copy number variants and annotated by ANNOVAR based on the refGene database. Calls were considered as deletions when total copy number was 0, and as amplifications when total copy number was at least 6.

Tumor mutation burden, microsatellite instability, and viral sequence analysis

Tumor mutation burden was calculated for each sample in terms of the number of non-synonymous variants per 1 Mb and scaled according to the exome panel size. Microsatellite instability (MSI) status was determined by MSIsensor [27] with a threshold of <10% for MSS (microsatellite stable), <10% and >30% for MSI-L (low), and >30% for MSI-H (high). To screen for viral integration events, unmapped reads were aligned against a sequence database of 198 human viruses (EBV, human papilloma virus, herpes simplex virus, among others).

Immunohistochemistry

Immunohistochemical (IHC) staining was performed with the Bond RX System (Leica Biosystems, Deer Park, IL, USA) using antibodies directed against MET (Spring Bioscience, Pleasanton, CA, USA, #M3440, clone SP44, 1:50) and CDK6 (Sigma-Aldrich Chemie GmbH, Taufkirchen, Germany, #HPA002637, 1:150). For all antibodies heat-induced antigen retrieval was carried out using the ER2 antigen retrieval solution for 20 min at pH 9.0 (Leica Biosystems). The immunostaining for EGFR (Epredia, #MS-378, clone 111.6, 1:100) was performed without heat pretreatment but with Bond

Enzym Pretreatment Kit (Leica Biosystems, #AR9551) for 5 min at 37 °C. Immunostaining for CDK6, EGFR and MET was evaluated based on the intensity of the stained tumor cells by a score of 0 (no staining), 1+ (weak staining), 2+ (moderate staining), and 3+ (strong staining). For MET and EGFR, only membranous staining was assessed. A positive MET status was defined by a combination of membranous MET immunostaining 2+ or 3+ with *MET*-amplification as described previously [10]. Immunostaining for CDK6 discriminated nuclear and cytoplasmic staining. In addition, the staining was described as homogeneous or heterogeneous to refer to the amount of stained tumor cells.

Analysis of BRAF, CDK, and MET amplification by droplet digital polymerase chain reaction

The amplification of *BRAF*, *CDK6*, and *MET* was studied by droplet digital polymerase chain reaction (ddPCR). DNA was extracted from FFPE tissue sections with the QIAamp DNA FFPE Tissue Kit (Qiagen) after microdissection of the immunohistochemically defined MET-positive and MET-negative tumor areas. The DNA concentration was measured by NanoDrop™ 2000c spectrophotometer (Thermo Fisher Scientific, Waltham, MA, USA) and PCRs were performed using 40 ng DNA per sample. Primers and probes were self-designed except of the described ones and custom-made from Biomers (Ulm, Germany) as follows: *CDK6*, forward primer 5′-CCTTCTCAGTAACACTTGGATC-3′, reverse primer 5′-ACTTCGGGTGCTCTGTAC-3′ and probe 5′-6-FAM-CGCTTGCCATTGCAGGTCGTCACGC-BMN-Q535-3′ and *MET*, forward primer 5′-CAATGTGAGATGTCTCCAGCAT-3′, reverse primer 5′-GGGAACTGATGTGACTTACCCTA-3′ [32] and probe 5′-6-FAM-ACGGACCCAATCATGAGCACTGC-BMN-Q535-3′. For the detection of the chosen

reference gene *RPP30* on chromosome 10 the forward primer 5′-GATTTGGACCTGCGAGCG-3′, reverse primer 5′-GCGGCTGTCTCCACAAGT-3′ and probe 5′-HEX-TCTGACCTGAAGGCTCTGCGCG-BMN-Q535-3′ [33] was used. All primers/probes were used at a concentration of 450/250 nM. For amplification analysis of *BRAF* we used validated copy number assays according to the instructions of the manufacturer (Bio-Rad, Feldkirchen, Germany, *BRAF*, #dHsaCP2500366 and *RPP30*, #dHsaCP2500350). The ddPCR was done using the ddPCR Supermix for Probes (No dUTP) (Bio-Rad), the QX200™ Droplet Generator (Bio-Rad), C100 Touch Thermocycler with DeepWell Reaction Module (Bio-Rad), and a QX200™ Droplet Reader (Bio-Rad). The ddPCR results were analyzed using the QuantaSoft™ Software version 1.7 (Bio-Rad).

Comparison of copy number variation data with data from cBioPortal

The cBioPortal for Cancer Genomics was used to verify (co-) amplifications of *BRAF*, *CDK6*, *EGFR*, and *MET* in public databases (date of analysis: July 16, 2024). We selected eight studies for visualization and analysis, which covered esophageal adenocarcinoma, stomach adenocarcinoma, adenocarcinoma of the gastroesophageal junction, esophagogastric adenocarcinoma, intestinal type gastric adenocarcinoma, tubular gastric adenocarcinoma, diffuse type gastric adenocarcinoma, mucinous adenocarcinoma, and papillary stomach adenocarcinoma [Memorial Sloan Kettering Cancer Center (MSK), 2020 [34]; MSK, Lancet Oncol 2020 [35]; MSK, J Natl Cancer Inst 2023 [36]; MSK, Clin Cancer Res 2022 [37]; TCGA, Nature 2017 [38]; MSK, Cancer Discovery 2017 [39]; Esophageal Adenocarcinoma, TCGA, PanCancer Atlas [38]; Stomach Adenocarcinoma, TCGA, PanCancer Atlas [12]]. Two different search criteria were applied: (1) prevalence of *BRAF*-, *CDK6*- and

EGFR amplification among *MET*-amplified cases (=*MET* pre-selected); (2) prevalence of *BRAF*-, *CDK6*-, *EGFR-,* and *MET* (co-) amplifications among all cases (=all-comers).

BRAF genotype

The *BRAF* genotype was obtained from a previous study, which was performed at the Department of Pathology, University Hospital Schleswig Holstein, Kiel, Germany [42].

References in supplementary material

10. Metzger ML, Behrens HM, Böger C*, et al.* MET in gastric cancer - discarding a 10% cutoff rule. *Histopathol*. 2016; **68**(2): 241.53.

12. Cancer Genome Atlas Research N. Comprehensive molecular characterization of gastric adenocarcinoma. *Nature*. 2014; **513**(7517): 202–9.

16. Okonechnikov K, Conesa A, Garcia-Alcalde F. Qualimap 2: advanced multi-sample quality control for high-throughput sequencing data. *Bioinformatics*. 2016; **32**(2): 292–4.

17. Lai Z, Markovets A, Ahdesmaki M*, et al.* VarDict: a novel and versatile variant caller for next-generation sequencing in cancer research. *Nucleic Acids Res*. 2016; **44**(11): e108.

18. Wang K, Li M, Hakonarson H. ANNOVAR: functional annotation of genetic variants from high-throughput sequencing data. *Nucleic Acids Res*. 2010; **38**(16): e164.

19. Fuentes Fajardo KV, Adams D, Program NCS*, et al.* Detecting false-positive signals in exome sequencing. *Hum Mutat*. 2012; **33**(4): 609–13.

21. Lek M, Karczewski KJ, Minikel EV*, et al.* Analysis of protein-coding genetic variation in 60,706 humans. *Nature*. 2016; **536**(7616): 285–91.

22. Tate JG, Bamford S, Jubb HC*, et al.* COSMIC: the Catalogue Of Somatic Mutations In Cancer. *Nucleic Acids Res*. 2019; **47**(D1): D941–D7.

23. Favero F, Joshi T, Marquard AM*, et al.* Sequenza: allele-specific copy number and mutation profiles from tumor sequencing data. *Ann Oncol.* 2015; **26**(1): 64–70.

24. Shinde J, Bayard Q, Imbeaud S*, et al.* Palimpsest: an R package for studying mutational and structural variant signatures along clonal evolution in cancer. *Bioinformatics*. 2018; **34**(19): 3380–1.

25. Rosenthal R, McGranahan N, Herrero J*, et al.* DeconstructSigs: delineating mutational processes in single tumors distinguishes DNA repair deficiencies and patterns of carcinoma evolution. *Genome Biol*. 2016; **17**: 31.

26. Talevich E, Shain AH, Botton T*, et al.* CNVkit: Genome-Wide Copy Number Detection and Visualization from Targeted DNA Sequencing. *PLoS Comput Biol*. 2016; **12**(4): e1004873.

27. Niu B, Ye K, Zhang Q*, et al.* MSIsensor: microsatellite instability detection using paired tumor-normal sequence data. *Bioinformatics*. 2014; **30**(7): 1015–6.

32. Oscorbin IP, Smertina MA, Pronyaeva KA*, et al.* Multiplex Droplet Digital PCR Assay for Detection of MET and HER2 Genes Amplification in Non-Small Cell Lung Cancer. *Cancers (Basel)*. 2022; **14**(6).

33. Sedlak RH, Hill JA, Nguyen T*, et al.* Detection of Human Herpesvirus 6B (HHV-6B) Reactivation in Hematopoietic Cell Transplant Recipients with Inherited Chromosomally Integrated HHV-6A by Droplet Digital PCR. *J Clin Microbiol*. 2016; **54**(5): 1223–7.

34. Sihag S, Nussenzweig SC, Walch HS*, et al.* Next-generation sequencing of 487 esophageal adenocarcinomas reveals independently prognostic genomic driver alterations and pathways. *Clin Cancer Res*. 2021; **27**(12): 3491–8.

35. Janjigian YY, Maron SB, Chatila WK*, et al.* First-line pembrolizumab and trastuzumab in HER2-positive oesophageal, gastric, or gastro-oesophageal

junction cancer: an open-label, single-arm, phase 2 trial. *Lancet Oncol*. 2020; **21**(6): 821–31.

36. Lumish MA, Walch H, Maron SB*, et al.* Clinical and molecular characteristics of early-onset vs average-onset esophagogastric cancer. *J Natl Cancer Inst*. 2024; **116**(2): 299–308.
37. Sihag S, Nussenzweig SC, Walch HS*, et al.* The Role of the TP53 pathway in predicting response to neoadjuvant therapy in esophageal adenocarcinoma. *Clin Cancer Res*. 2022; **28**(12): 2669–78.
38. Cancer Genome Atlas Research N, Analysis Working Group: Asan U, Agency BCC*, et al.* Integrated genomic characterization of oesophageal carcinoma. *Nature*. 2017; **541**(7636): 169–75.
39. Janjigian YY, Sanchez-Vega F, Jonsson P*, et al.* Genetic Predictors of Response to Systemic Therapy in Esophagogastric Cancer. *Cancer Discov*. 2018; **8**(1): 49–58.
42. Warneke V, Behrens HM, Haag J*, et al.* Prognostic and putative predictive biomarkers of gastric cancer for personalized medicine. *Diagnostic Mol Pathol*. 2013; **22**(3): 127–37.
56. Tarasov A, Vilella AJ, Cuppen E*, et al.* Sambamba: fast processing of NGS alignment formats. *Bioinformatics*. 2015; **31**(12): 2032–4.
57. Mose LE, Wilkerson MD, Hayes DN*, et al.* ABRA: improved coding indel detection via assembly-based realignment. *Bioinformatics*. 2014; **30**(19): 2813–5.

[* references 56.57 only in supplementary file]

Supplementary Figures

a **case D1_TP53_forward:**
mut. c.437G>A, hg19: g.7578493 C>T TGG>TAG (p.W146X)

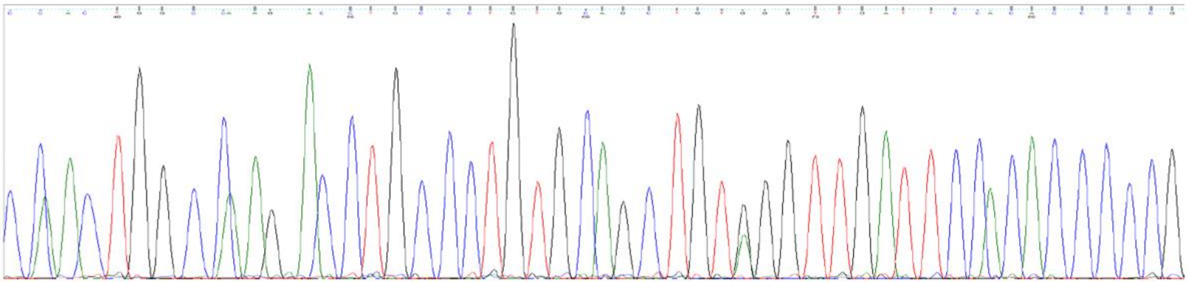

b **case D2 _TP53_forward:**
mut. c.743G>A, hg19: g.7577538 C>T CGG>CAG (p.R248Q)

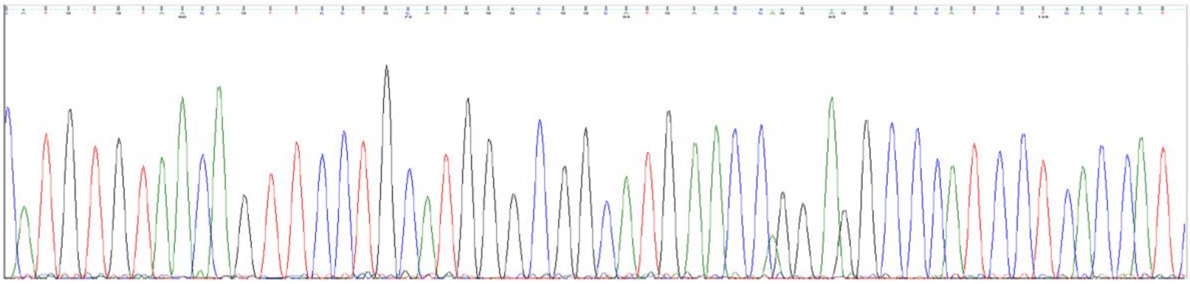

Figure S1. *TP53* mutations in the two discovery cases. *TP53* mutations detected in whole-exome sequencing were confirmed by Sanger sequencing, case D1 (A) and case D2 (B).

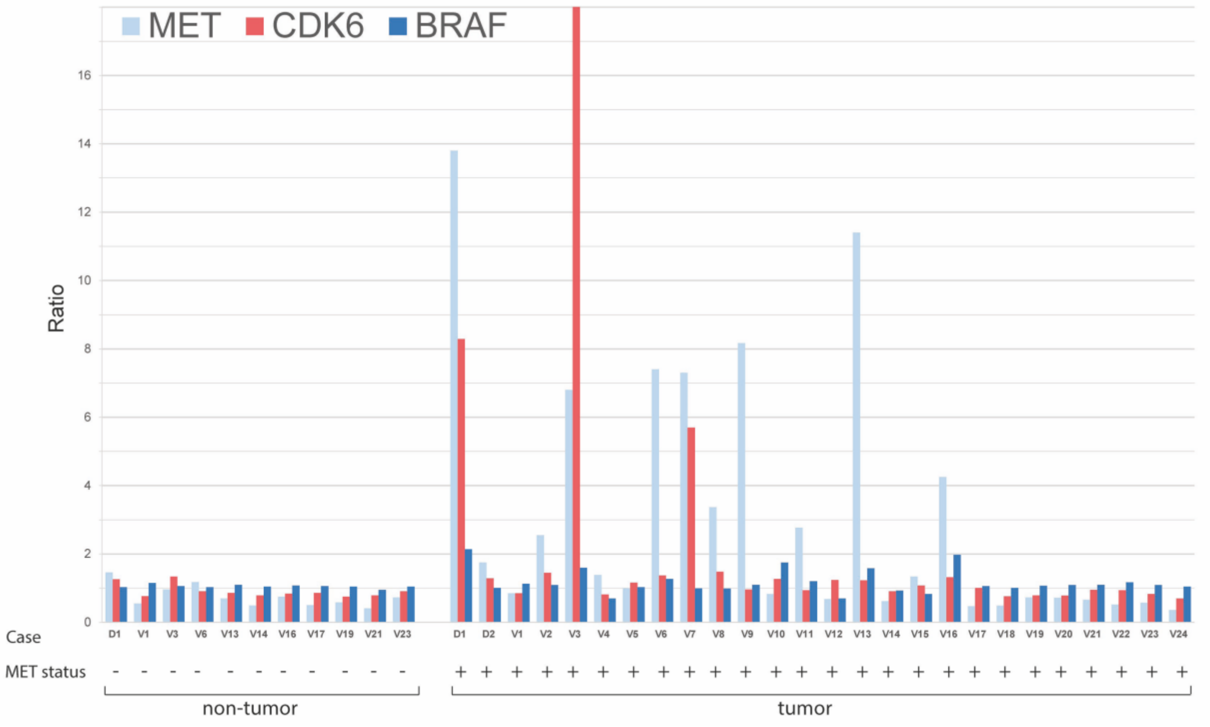

Figure S2. Intertumoral heterogeneity of gene amplification(s). Evaluation of gene amplification in 2 cases of the discovery cohort and 24 cases of the validation cohort of *MET*-amplified gastric cancer assessed by digital droplet PCR. Only immunohistochemically MET-positive tumor areas were applied. The ratio of the amount of target genes on chromosome 7 was calculated in relation to the amount of *RPP30* gene on chromosome 10. Non-tumor DNAs from 11 cases served as negative controls.

BRAF = b-Raf proto-oncogene; CDK6 = cyclin dependent kinase 6; MET = MET proto-oncogene; RPP30 = ribonuclease P/MRP subunit p30.

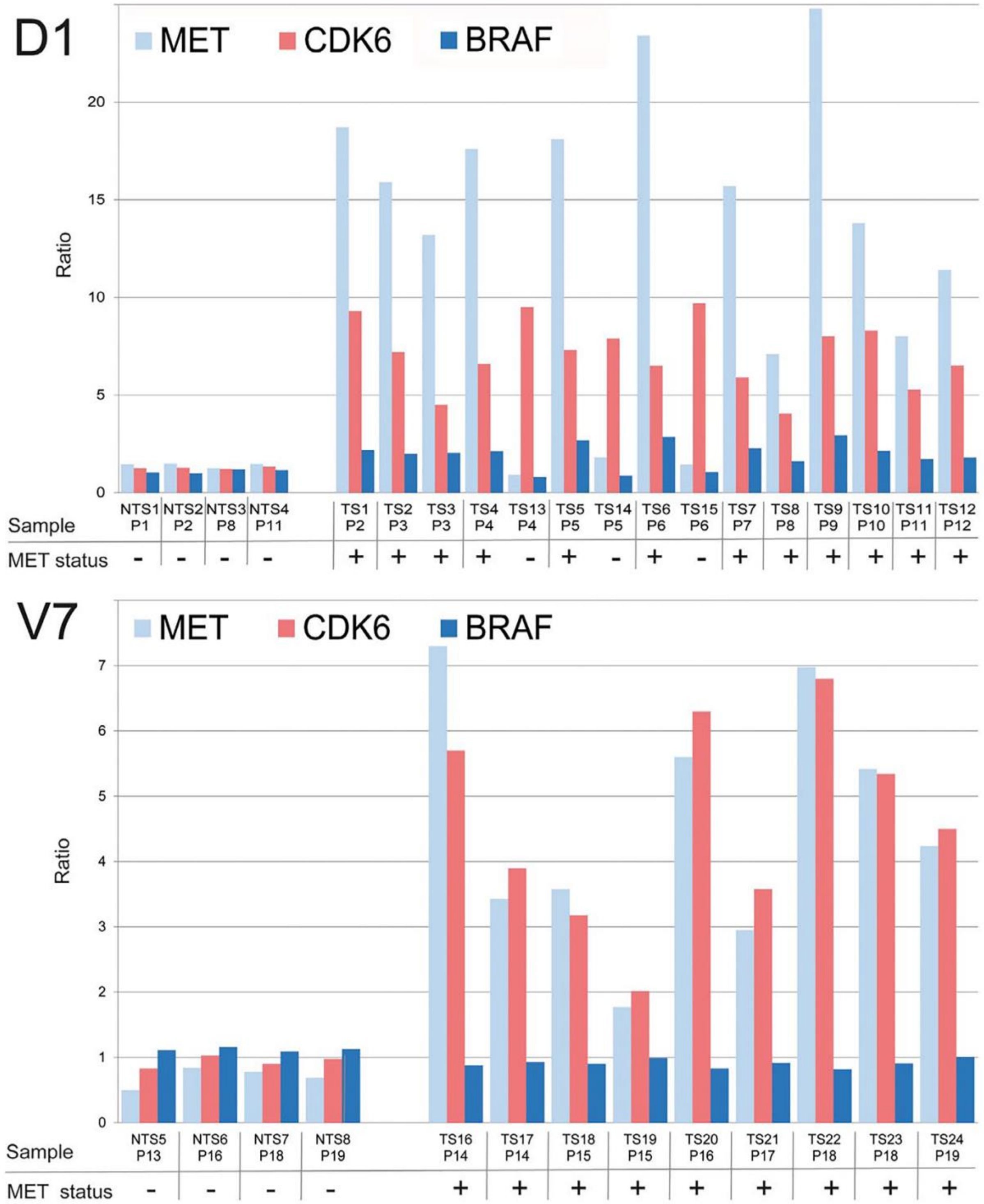

Figure S3. Intratumoral heterogeneity of gene amplification(s). Samples obtained from 17 tumor bearing and 2 non-tumor bearing paraffin blocks obtained from 2 cases (D1 and V7) were forwarded to digital droplet PCR. Together 32 microdissected samples were prepared and subjected to 96 ddPCR analysis, eight samples from non-neoplastic mucosa (2 × 4 from each case) which served as controls (NTS) and 24 [15 (D1) and 9 (V7)] from 17 tumor-bearing paraffin blocks [11 (D1) and 6 (V7) named TS = tumor-samples]. From 7 tumor-bearing paraffin blocks 2 different tumor areas were assessed separately, for example, in case D1 from MET-positive and -negative tumor areas (TS4, TS5, TS6 and TS13, TS14, TS15). The ratio of the amount of target genes on chromosome 7 was calculated in relation to the amount of *RPP30* gene on chromosome 10. NTS = non-tumor sample; TS = tumor sample; P = paraffin block; BRAF = b-Raf proto-oncogene; CDK6 = cyclin dependent kinase 6; MET = MET proto-oncogene; RPP30 = ribonuclease P/MRP subunit p30.

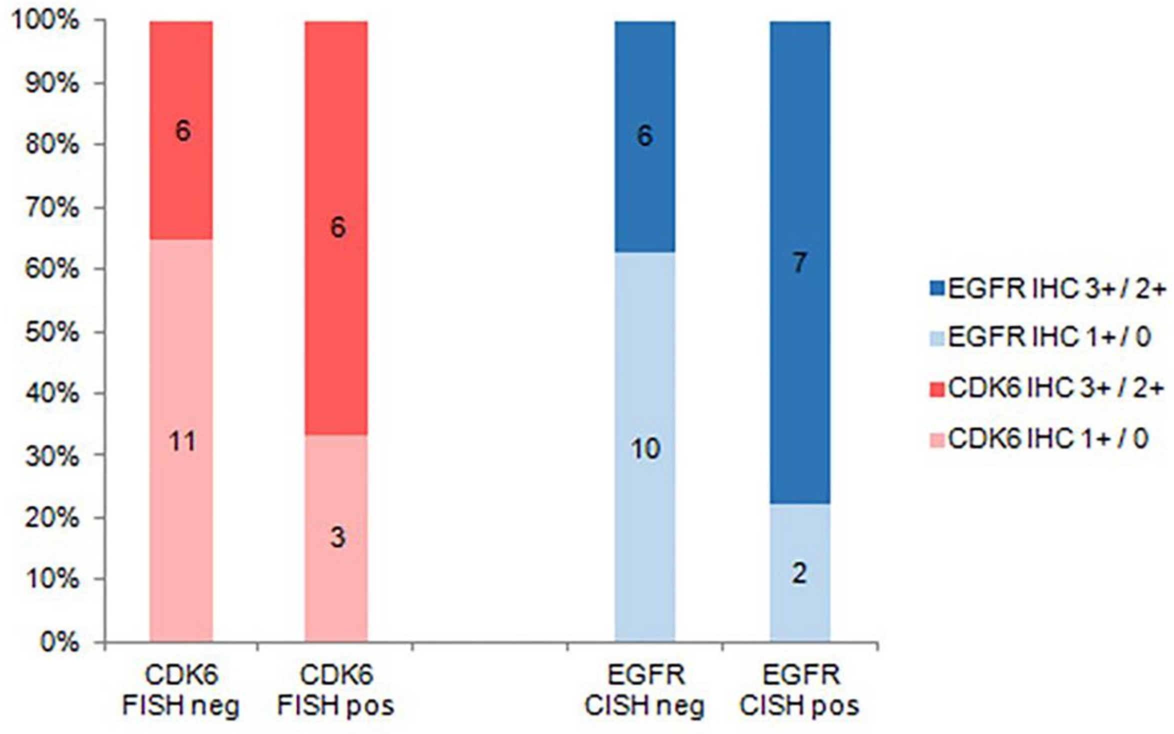

Figure S4. Graphical illustration of the correlation of immunostaining with gene amplification for *CDK6* and *EGFR*. Numbers are cases.
CDK6 = cyclin dependent kinase 6; EGFR = epidermal growth factor receptor; CISH = chromogenic in situ hybridization; FISH = fluorescence in situ hybridization; IHC = immunohistochemistry.
